# Disinfection of football protective equipment using chlorine dioxide produced by the ICA TriNova system

**DOI:** 10.1186/1471-2458-9-326

**Published:** 2009-09-08

**Authors:** Anthony L Newsome, John D DuBois, Joel D Tenney

**Affiliations:** 1 Department of Biology, Middle Tennessee State University, Murfreesboro, TN, USA; 2 ICA TriNova, LLC, 24 Woodland Trail, Newnan, GA, USA

## Abstract

**Backround:**

Community-associated methicillin-resistant *Staphylococcus aureus *outbreaks have occurred in individuals engaged in athletic activities such as wrestling and football. Potential disease reduction interventions include the reduction or elimination of bacteria on common use items such as equipment. Chlorine dioxide has a long history of use as a disinfectant. The purpose of this investigation was to evaluate the ability of novel portable chlorine dioxide generation devices to eliminate bacteria contamination of helmets and pads used by individuals engaged in football.

**Methods:**

In field studies, the number of bacteria associated with heavily used football helmets and shoulder pads was determined before and after overnight treatment with chlorine dioxide gas. Bacteria were recovered using cotton swabs and plated onto trypticase soy agar plates. In laboratory studies, *Staphylococcus aureus *was applied directly to pads. The penetration of bacteria into the pads was determined by inoculating agar plates with portions of the pads taken from the different layers of padding. The ability to eliminate bacteria on the pad surface and underlying foam layers after treatment with chlorine dioxide was also determined.

**Results:**

Rates of recovery of bacteria after treatment clearly demonstrated that chlorine dioxide significantly (p < 0.001) reduce and eliminated bacteria found on the surface of pads. For example, the soft surface of shoulder pads from a university averaged 2.7 × 10^3 ^recoverable bacteria colonies before chlorine dioxide treatment and 1.3 × 10^2 ^recoverable colonies after treatment. In addition, the gas was capable of penetrating the mesh surface layer and killing bacteria in the underlying foam pad layers. Here, 7 × 10^3 ^to 4.5 × 10^3 ^laboratory applied *S. aureus *colonies were recovered from underlying layers before treatment and 0 colonies were present after treatment. Both naturally occurring bacteria and *S. aureus *were susceptible to the treatment process.

**Conclusion:**

Results of this study have shown that chlorine dioxide can easily and safely be used to eliminate bacteria contamination of protective pads used by football players. This could serve to reduce exposure to potential pathogens such as the methicillin-resistant *Staphylococcus aureus *among this group of individuals.

## Backround

Outbreaks of skin infections among individuals engaged in sports activities is an emerging health issue. The methicillin-resistant *Staphylococcus aureus *(MRSA) have recently garnered much of the attention in both the popular media and among health care professionals. The general population is susceptible to MRSA outbreaks. Individuals engaged in athletic events in which the participants are in close contact or share equipment, however, may be at additional risk [1-3]. There continues to be some speculation regarding the risk factors, modes of transmission, means of intervention, and potential preventative measures [1,2].

In efforts to reduce risk, it has been suggested outbreak intervention measures include disinfection of showers and whirlpools, elimination of towel sharing, increased frequency of cleaning facilities and athletic gear, and overall discouraging the sharing of personal items [1,2]. The barring of case-players from playing unless wounds are covered has also been a suggested preventative measure. MRSA outbreaks have recently been linked directly to organized football programs from the high school level to that of professional athletes [1-3].

The disinfection of large bulky items such as football shoulder pads and helmets are not readily accomplished. For an entire team it would be both time consuming and difficult to manually wipe and disinfect all surfaces on the equipment. Even this might not ensure the elimination of potential bacteria pathogens, especially those that reside beneath the mesh covering on pads. Professional services are available that afford the opportunity to refurbish and cleanse the pads but the cost of these services may be beyond the means of many athletic programs.

Chlorine dioxide (ClO_2_) gas has a long history of use as a disinfectant. It is used worldwide for the treatment of potable water, for sanitation food preparation, and for bleaching pulp in the paper industry [4,5]. The gas is too unstable to be shipped and must be prepared at the location where it is applied. Historically, this has often required the use of dedicated equipment and the training of personnel. Advances in ClO_2 _generation technology have provided the ability to produce small amounts of the gas to meet a variety of specific needs. One system recently developed by ICA TriNova (Newnan, GA.) consists of a two part impregnate encased within a sachet that is gas permeable but water impermeable. When submerged in water, gaseous ClO_2 _is released creating a bactericidal solution. This system does not require humidity or moisture for activation. In-vitro tests from this laboratory have demonstrated that small levels of ClO_2 _locally produced by these sachets can have a rapid bactericidal effect against vegetative bacterial cells, bacteria spores, and amoebae [6,7]. It has been demonstrated that aqueous ClO_2 _generated using ICA TriNova gas generation system was effective against a variety of bacteria (*Listeria, Pseudomonas, Salmonella, Yersinia *and *Staphylococcus*), yeasts, and molds applied to blueberries [8]. The highest log reduction (4.56 log cfu/g) was achieved with *S. aureus *using 15 ppm of ClO_2 _for 30 min. Gaseous ClO_2 _produced by different systems has also been the focus of other researchers investigating sanitizers for use in the food industry (reviewed in 8). Research into the antimicrobial mechanism of ClO_2 _has been directed at the identification of specific chemical reactions between ClO_2 _and biomolecules and evolution of the effect of ClO_2 _on physiological functions. Chlorine dioxide appeared to react with the amino acids cysteine, tryptophan, and tyrosine in viral agents, but the primary molecular target remained unclear. The primary site of action of ClO_2 _on bacteria was not believed to be the dehydrogenase enzymes, the protein synthesizing complex, or DNA. It was suggested that disruption of the organisms outer membranes or gross change in membrane permeability contributed to a lethal effect [9].

The purpose of this investigation was to examine the potential of the ICA TriNova ClO_2 _gas generation system to significantly reduce or eliminate culturable bacteria from the surface of heavily used football pads (shoulder pads and helmets) and to likewise diminish bacteria that reside beneath the mesh covering and in the underlying foam padding of pads. In the current study, test results suggested that this ClO_2 _generation system was effective in the significant reduction and in some cases the elimination of bacterial contamination of used equipment.

## Methods

### Field trial studies

Shoulder pads and helmets (in current use) from a local high school (14 sets) and university (15 sets) were used in the field study. Informed consent for this study was given by personnel in the athletic departments. Prior to testing, the pads appeared to be well maintained. Typical maintenance included spot cleaning for removal of visible debris and repair of damaged areas such as straps or torn areas. This was performed by an equipment manager at each institution. A hard outer surface of shoulder pads (plastic covering over the shoulder area) and a soft mesh layer (inner surface that rests against the chest area) were sampled for the presence of bacteria immediately prior to treatment with ClO_2_. For this, an area of approximately 50 cm^2 ^was vigorously rubbed with a sterile cotton swab that was then rubbed over the surface of a trypticase soy agar plate (TSA). The soft inner lining of helmets was sampled in a similar manner. A single shoulder pad and helmet were placed in a 113 L (30 gallon) plastic garbage bag purchased from a local store. For ClO_2 _treatment, a ICA TriNova sachet capable of generating approximately 500 mg of ClO_2 _overnight was placed in the bag and the bag sealed by tying a knot in the end. Chlorine dioxide treatment was allowed to continue overnight (12 to 14 h). Equipment was removed from the bag and an area on the surface of each equipment item adjacent to the pre-treatment site was sampled and platted onto TSA in an identical manner. Agar plates were incubated at 37°C for 48 h and the relative number of colony forming units (CFU) were determined.

### Application and recovery of *Staphylococcus aureus *applied to football pads

To assess the effects on *Staphylococcus aureus *that potentially might contaminate inanimate objects such as pads, lab cultures of the bacteria were applied to the pad surface and subsequently treated with ClO_2 _gas. The gas also served to reduce or eliminate laboratory applied *S. aureus *bacteria on the pad surface and in the underlying foam pad layer. *Staphylococcus aureus *(ATCC 25923) was cultured (37°C) overnight in tubes containing 9 ml of trypticase soy broth. Cells were harvested by centrifugation (500 × g for 30 min) and suspended in sterile saline (8.5 g NaCl/L). The padded portion of 3 sets of shoulder pads was removed and autoclaved so that contaminating bacteria were eliminated. An area of 25 cm^2 ^was marked off on pads using masking tape. Suspensions of *S. aureus *(typically 1 ml in volume) were applied to the inner mesh surface of a portion of shoulder pads using a sterile paint brush and allowed to absorb onto the pad surface overnight. A 100 ul aliquot of the original bacteria suspension that was applied to the pad surface was also serially diluted in saline and plated onto TSA plates to determine the number of bacteria added to the pads before treatment. Controls consisted of pads similarly treated with bacteria but not subject to the ClO_2 _treatment. Following exposure to chlorine dioxide, the mesh surface was rubbed with a sterile cotton swab and then rubbed onto the surface of a Mannitol Salt Agar plate (this medium was selective for *S. aureus *due to its high salt content). Bacteria fermentation of mannitol results in a pH change that turns the medium from red to yellow. Using sterile razor blades and forceps a 2.5 × 2.5 cm portion of the pad was cut away. The mesh covering was pealed back and the underside portion of the mesh covering was rubbed onto the surface of a Mannitol Salt Agar plate. Next, the outermost portion of the foam padding was rubbed onto the surface of another Mannitol Salt Agar plate. The outermost portion of the foam padding was then cut away and an inner portion of foam (0.5 cm deep) rubbed onto an additional Mannitol Salt Agar plate.

### Treatment of spore coupons enclosed in Tyvek

Small steel discs impregnated with 10^3 ^(total number of spores) spores of *Bacillus atrophaeus *enclosed in Tyvek (Apex Laboratories, Apex, N.C., USA) were in some instances placed alongside the football pads during the treatment process. Biological indicators such as this have been used to assess the generation of sterilizing conditions for a variety of antimicrobial systems. After exposure to ClO_2 _gas, the Tyvek covering was opened and the steel disc removed using aseptic technique. The steel disc was placed into 7 mL of trypticase soy broth (TSB) and incubated for 72 h at 35°C. Any surviving spores germinated and growth was easily determined by visual observation of the tubes containing TSB.

### Statistical analysis

Data were analyzed using the Student t-test, Paired Measures t-test and their nonparametric equivalents of the Mann-Whitney U-test and the Wilcoxon test. All statistical analyses were performed using SigmaStat Statistical Software (SPSS Inc.).

## Results

### Field trial studies

Results showing the reduction or total elimination of recoverable bacteria (CFU) from chlorine dioxide treated football pads and helmets from a local high school are outlined in Table 1. For this, 14 different treatment sets were examined and each treatment set consisted of one shoulder pad and helmet. Before treatment the number of recoverable CFU on the soft surfaces was often greater than 300 colonies on agar plates. These plates were designated as TNTC (too numerous to count). Following treatment it was apparent that the number of recoverable CFU was significantly reduced (p < 0.001; Table 1). In a subsequent field trial study (Table 2), 15 sets of football pads and helmets from a local university were assessed and treated in the same fashion. Here an attempt was made to enumerate bacteria on plates containing more than 300 colonies. Plates were placed on a grid and the number of bacteria within 5 grids was determined. This number was averaged and extrapolated to give an estimate of the number of bacteria on plates. In this case we were able to determine that shoulder pad hard surfaces averaged 80 CFU before treatment and 7 CFU after treatment. Shoulder pad soft surfaces averaged 2,686 CFU before treatment and 133 CFU after treatment. Helmet soft surfaces averaged 20 CFU before and less than 1 CFU after treatment. Once again bacteria were totally eliminated or significantly reduced (Table 2).

**Table 1:** Recovery of bacteria colonies (numbers represent colony count units) from used high school football pads before and after treatment with chlorine dioxide.

	**Shoulder Pad-Hard Surface**		**Shoulder Pad-Soft Surface**		**Helmet-Soft Surface**	
	
**Set**	**Before**	**After**	**Before**	**After**	**Before**	**After**
1	50	0	TNTC*	0	30	0
2	300	0	400	0	50	0
3	200	2	TNTC	NA	100	0
4	200	1	TNTC	0	20	0
5	200	0	300	0	30	0
6	100	1	TNTC	50**	100	NA
7	100	0	TNTC	0	50	0
8	50	2	TNTC	50**	TNTC	1
9	50	0	TNTC	1	30	3
10	50	0	TNTC	0	150	1
11	75	1	200	10	50	1
12	TNTC	0	TNTC	0	50	1
13	200	0	TNTC	0	100	2
14	75	0	50	5	100	5

P***	p < 0.001		p < 0.001		p < 0.001	

*TNTC those plates that had greater than 300 colonies present

** The greater number of recoverable bacteria was most likely a reflection of pad placement such that ClO_2 _gas did not get good access to the treatment site.

*** Mann-Whitney U-Test

**Table 2:** Recovery of bacteria colonies (numbers represent colony count units) from used university football pads before and after treatment with chlorine dioxide.

	**Shoulder Pad-Hard Surface**		**Shoulder Pad-Soft Surface**		**Helmet-Soft Surface**	
	
**Set**	**Before**	**After**	**Before**	**After**	**Before**	**After**
1	20	0	800	5	3	3
2	100	3	5,600	500	50	0
3	40	4	4,000	40	1	1
4	200	5	300	50	3	0
5	20	0	5,600	500	5	1
6	20	3	500	150	5	0
7	20	0	200	2	1	0
8	200	0	800	30	10	0
9	50	1	5,600	150	150	1
10	300	5	1,400	15	0	0
11	30	1	5,600	2	40	1
12	60	1	1,600	40	10	1
13	30	2	1,100	4	10	1
14	60	30	1,600	15	10	1
15	50	50*	5,600	500	10	1

t-test	p = 0.003		p < 0.001		p = 0.056	
U-test	p < 0.003		p < 0.001		p < 0.00	

* The greater number of recoverable bacteria was most likely a reflection of pad placement such that ClO_2 _gas did not get good access to the treatment site.

### Survival of *Staphylococcus aureus *on football pads

In 3 different *S. aureus *applications, the number of bacteria applied to the pad surface was varied from 10^8 ^to 6.5 × 10^8 ^(the amount present in a 1 ml application volume). When laboratory cultures of *S. aureus *were applied to sterile pads that had been autoclaved prior to bacteria application, the number of recoverable bacteria decreased over time. Large numbers of bacteria, however, were still recovered at 168 h after application of bacteria. Results of a typical application and recovery experiment are given in Table 3. It was apparent that the greatest number of recoverable bacteria were associated with the underside of the mesh layer and the top portion of the foam padding.

**Table 3:** Application (10^8 ^CFU) and recovery of *Staphylococcus aureus *from football pads over time (numbers represent colony count units).

**Time**	**Surface mesh swab**	**Under Mesh**	**Top of foam pad**	**0.5 cm into foam pad**
24 h	2,296	22,640	30,680	1,904
48 h	222	17,104	20,664	343
120	27	8,912	12,136	105
168 h	14	5,320	6,216	26

### Treatment of pads inoculated with *S. aureus*

When 1 ml of a *S. aureus *suspension was applied to pads, the recovery of bacteria was significantly (p = 0.029) reduced after treatment with chlorine dioxide gas. For these tests, the number of bacteria applied to pads was varied from 3.0 × 10^8 ^CFU to 1.3 × 10^9 ^CFU. Treatment times were 5 h, 10 h, and 24 h. In each instance, no *S. aureus *could be recovered from pads after a 5 h treatment. Results from a typical treatment are outlined in Table 4. Included in each of these tests also were steel disks impregnated with 10^3 ^spores of *Bacillus atrophaeus*. The steel disks were placed adjacent to the pads undergoing ClO_2 _treatment. After the 5 h treatment there were no outgrowth of spores from the steel disks.

**Table 4:** Application (1.3 × 10^8 ^CFU) and ClO_2 _treatment (5 h) of *Staphylococcus aureus *applied to football pads (numbers represent colony count units).

	**Surface mesh swab**	**Under Mesh**	**Top of foam pad**	**0.5 cm into foam pad**
5 h (treated)	0	0	0	0
Control (untreated)	3,528	7,056	7,056	4,536

t-Test:	p = 0.029			

3 of 3 spore strips (10^3 ^spores of *Bacillus atrophaeus *on steel discs enclosed in Tyvak) had no growth in TSB.

### Effect of pre-treatment of football pads with ClO_2 _and subsequent recovery of bacteria following 7 days use

The potential of chlorine dioxide treatment to subsequently diminish bacteria colonization on pads was investigated using *S. aureus *in a lab setting and assessing the presence of bacteria on used pads after ClO_2 _treatment. For laboratory studies, one set of shoulder pads was treated overnight with ClO_2 _and a second set of pads served as an untreated control. A 1 ml suspension containing 6.5 × 10^8 ^*S. aureus *was applied to each of the pads and allowed to sit overnight at room temperature. Subsequently, there was no significant difference in the recovery of S. *aureus *from the top mesh covering, underside of the mesh, or within the foam padding.

In a field trial study, 3 sets of shoulder pads were treated overnight with ClO_2 _and an additional 3 sets of pads served as untreated controls. Results showing the number of recoverable bacteria from pre-treatment, post-treatment, and after 7 days use are outlined in Table 5. Although not statistically significant, there was a reduction in the number of recovered bacteria observed in this study.

**Table 5:** Effect of pre-treatment of football pads with ClO_2 _and its impact on subsequent recovery of bacteria following 7 days use (numbers represent colony count units).

	**pre-treatment**	post-treatment*	**7 days use**
Control pads (untreated)			

1	296	290	18,648
2	349	138	24,696
3	1,120	1,176	3,528

ClO_2 _Treated pads			

1	2,296	337	2,464
2	274	9	324
3	105	15	61

t-Test	p = 0.708	p = 0.292	p = 0.082

For this 3 different control (untreated pads) and 3 different treated pads (treated with ClO_2) _were evaluated.

*Here post-treatment was a control group and there was no treatment, but a second sample was taken and evaluated for colony count units.

## Discussion

Results demonstrated that bacteria were readily cultured from used shoulder pad and helmet surfaces. It was apparent the greatest number of recoverable bacteria was associated with the soft mesh surfaces on shoulder pads. Typically, much fewer bacteria were recovered from the soft padding of helmets. This padding was much less porous than the mesh covering over the soft foam padding, and it, in part, may account for the lower number of bacteria from helmet pads.

There is a paucity of knowledge about the relative occurrence and the degree to which protective football pads are colonized by bacteria. This lack of knowledge may be of increasing significance since skin infections by bacteria (such as *S. aureus*) among athletes continues to be an emerging issue. It is believed many of these infections due to *S. aureus *are likely the result of skin to skin contact. However, it is also likely that the environment itself can play a role in the dissemination of bacteria dermal pathogens. It is also now apparent that our knowledge is lacking about the potential of sports equipment to play a role in the dissemination of bacteria that might cause.

One aim of this study was to document the degree to which football pads might be colonized by bacteria. The pre-chlorine dioxide treatment sampling demonstrated that relatively large numbers of bacteria can be recovered from rubbing a cotton swab over a small portion (50 cm^2^) of a pad surface. It was also apparent that the bacteria were not evenly distributed over the pad surfaces. The recovery of bacteria from the soft mesh surfaces was typically greater than that from hard plastic surfaces. It could be hypothesized that this might be due to the more porous mesh surface of soft pads. The porous surfaces could also entrap moisture and debris which support bacteria nutrition and viability. These results suggest also the colonization on soft pad surfaces could be affected by modification of the mesh covering to more closely approximate that found on the hard surfaces. This notion is supported by the lower number of bacteria recovered from soft surfaces of the helmet lining. Here, the soft pad covering was much less porous than the mesh covering of shoulder pads. Although this was a padded surface, the number of bacteria recovered was similar to that seen with the hard plastic surfaces of the shoulder pads.

The ability of chlorine dioxide treatment to totally eliminate or significantly diminish the recovery of bacteria from hard and soft pad surfaces was apparent. Exactly why in some instances recoverable bacteria were totally eliminated and in other instances a small fraction of the pre-treatment value was recoverable is not known. This could most likely be explained, however, by the gas not gaining access to areas in which some bacteria were recovered. This is likely a reflection of some pads being placed in a manner such that the gas was not as accessible to certain sites. This would suggest that in future treatments the pads should be placed in the bag enclosure in a specific manner to ensure that the gas is equally accessible to all areas. In the present study, placement of pads in enclosures was not standardized and this likely accounted for some variation in recovery of bacteria. Another potential contributing factor is that in some instances the pre-treatment recoverable bacteria values were exceptionally high, thus contributing to the potential some bacteria might survive the chlorine dioxide treatment.

Bacteria spores are much more resistant to disinfectants than the vegetative forms. The ability to kill the spores of bacteria in the genus *Bacillus *is a commonly used method to assure sterilizing conditions are generated in a variety of microbial treatment processes. Therefore the ability of the treatment process to consistently kill 10^3 ^spores of *Bacillus atrophaeus *enclosed in Tyvek additionally demonstrates that a potent bactericidal effect was generated.

It was also relevant to this study to determine the effect of ClO_2 _on specific bacteria such as the medically important *Staphylococcus aureus*. Initial studies showed that *S. aureus *survived on pads for at least 168 h although as might be expected the number of recoverable bacteria decreased over time. Of additional potential importance was the observation that the greatest number of bacteria were recovered not from the pad surface where the bacteria were applied, but rather on the underside of the mesh cover and into the underlying foam pad. It is possible that the wicking action of the pad covering served to carry *S. aureus *into the pad. Here the bacteria likely were not as subject to drying effects which could diminish the number of viable bacteria. These results suggested that in any bactericidal system used to eliminate bacteria, consideration be given to the ability of the bactericidal agent to penetrate the surface of the pad covering. Despite the occurrence of *S. aureus *below the pad surface they were susceptible to the bactericidal effects of chlorine dioxide gas after treatments as short as 5 h.

Initial studies using laboratory cultures of *S. aureus *suggested that pre-treatment of pads with chlorine dioxide failed to diminish subsequent colonization by bacteria. However, in field studies at a local college with used pads it was apparent that when pads were used on a daily basis a pre-treatment protective effect could be observed. The difference in the these two observations can most likely be explained by the fact that in laboratory studies with *S. aureus *an extremely large inoculating dose was used. It is possible this may have overwhelmed any protective effect that might have been present. In the field studies it was apparent that much lower numbers of bacteria were encountered that a protective effect was able to be expressed and detected using our recovery methods.

The first methicillin-resistant *S. aureus *(MRSA) was reported in England in 1961 shortly after the antibiotic was put into clinical use [10]. By 1968, it was reported in Japan, Europe, Australia, and the United States [11]. Hospital acquired (nosocomial) MRSA infections have long been an important issue in health care facilities. Here it has been demonstrated that the hospital environment frequently becomes contaminated, thus serving as a potential source of infection [12-16]. In addition, potential pathogens such as MRSA may survive long periods on surfaces [16]. *Staphylococcus aureus *survived 56 days on polyster and more than 90 days on polyethylene plastic. A larger inoculum size promoted increased survival time but even a few hundred bacteria survived for days on fabrics. There was no relationship between antibiotic resistance and survival among the different *S. aureus *studied [17]. Recent studies suggest on site equipment, supplies, and health care facilities should be disinfected to promote reduction of nosocomial MRSA infections [12-15,17]. It can be postulated how these materials could serve as a vector for the spread of *S. aureus *at least in the hospital environment. The survival time of *S. aureus *on materials denotes the potential importance of through disinfection of hospital fabrics and plastics to minimize exposure [13,17].

In the last 10 years MRSA has emerged within the community (community acquired MRSA or CA-MRSA). The more widespread occurrence of CA-MRSA (especially among individual engaged in certain athletic activities) was noted in the 1990's. Affected individuals frequently lacked the risk factors associated with hospital-associated (nosocomial) MRSA. The current substantial increase in community-acquired MRSA provides a challenge in selecting appropriate intervention strategies. This is additionally complicated by the report that the USA 300-0114 strain of *S. aureus *is primarily responsible for community-acquired MRSA in the United States and its natural reservoir remains an open question [18].

Studies have suggested the *de novo *development of MRSA when a genetic element (staphylococcal cassette chromosome mec) is acquired by methicillin-susceptible *S. aureus*. This transfer has occurred only a few times. It was postulated the worldwide emergence of MRSA was the result of dissemination of only a few clonal types [12]. Likely, most all patients with MRSA infection or colonization have acquired their MRSA strain from an external source. Therefore control efforts must in large part, focus on preventing transmission. This concept is supported by the observation that MRSA can be successfully controlled with infection control practice. The MRSA strains did not exhibit increased resistance to commonly used disinfectants. This has been true also for a variety of antibiotic resistant gram-negative and gram-positive bacteria [12]. This provides additional support for the concept that gaseous chlorine dioxide has excellent potential to eliminate bacteria such as the MRSA from sports equipment.

## Conclusion

Whether environmental decontamination of materials can serve to reduce outbreaks of community-acquired MRSA in a manner comparable to that of nosocomial MRSA will require additional studies. Nevertheless, the results of this study demonstrated current technology is available to easily and economically decontaminate shared equipment used by one group of individuals (football players) that appear to be at greater risk of community-acquired MRSA than the general population. The chlorine dioxide generation system described in this report could serve as a good device for use in future studies to define the role of environmental decontamination in the reduction of community-acquired MRSA.

## Competing interests

The chlorine dioxide generation sachets used in this study were provided by ICA TriNova. The author JDT is a member of this organization. The first author (ALN), has in the past received material and financial support from ICA TriNova for research studies directed at chlorine dioxide. The 2^nd ^author (JDD) has no competing interests.

## Authors' contributions

JDT was responsible for treatment protocols and contributed to the experimental design. JDD contributed to data interpretation and was responsible for statistical analysis. ALN Contributed to culture protocol and assessment of results. All authors have read and approved the final version.

## Pre-publication history

The pre-publication history for this paper can be accessed here:


